# Acetabular Reconstruction in a Rare Case of Ligamentum Teres Tenosynovial Giant Cell Tumour (TGCT) Causing Extensive Destruction

**DOI:** 10.7759/cureus.52171

**Published:** 2024-01-12

**Authors:** Georgios Douvlis, Christothea–Alexandra Tsiridis, Zakareya Gamie, Antonia Mpintoudi, Nikolaos Milonakis, Eustathios Kenanidis, Eleftherios Tsiridis

**Affiliations:** 1 Department of Academic Orthopaedics, Papageorgiou General Hospital, Aristotle University of Thessaloniki, Thessaloniki, GRC; 2 Department of Medicine, Institute of Medical and Biomedical Education, St. George’s University of London, London, GBR; 3 Department of Interventional Radiology, Bioitatriki Clinic, Thessaloniki, GRC

**Keywords:** tenosynovial giant cell tumour, pigmented villonodular tendosynovitis(pvns), acetabular reconstruction, ligamentum teres, cementless total hip arthroplasty

## Abstract

Tenosynovial giant cell tumour (TGCT), previously called pigmented villonodular tenosynovitis (PVNS), is a rare benign, locally aggressive condition that primarily affects the synovial lining of large joints, such as the knee, the hip, and the ankle. TGCT of the hip joint is a relatively scarce entity, and its diagnosis is often challenging. This article reports a case of TGCT affecting the left acetabulum, the left femoral head, and the ligamentum teres of the hip joint in a 39-year-old woman who presented to our clinic three months after the onset of symptoms. The patient underwent a biopsy, computer tomography (CT), and magnetic resonance imaging (MRI). All tests were inconclusive. Total hip arthroplasty (THA) was subsequently performed, leading to healing of the lesion previously present. Following surgery, a second biopsy classified this lesion as TGCT. By sharing our experience with this rare manifestation, we aim to contribute to the growing body of knowledge on the diagnosis and management of TGCT, specifically when it occurs in the hip joint.

## Introduction

Tenosynovial giant cell tumour (TGCT) is the updated term used in the 2020 WHO Soft Tissue and Bone Tumours Classification (5th ed.) for pigmented villonodular synovitis. TGCTs can be categorised based on their location and growth patterns. TGCT is a neoplastic-like condition of the synovium, characterised by the proliferation of synovial cells and the presence of hemosiderin-laden macrophages. TGCT is considered a monoarticular disease [1,2]. It most commonly involves the knee joint, while other sites such as the hip joint are less frequently affected posing diagnostic dilemmas. Due to its low incidence, TGCT is poorly understood. The aetiology of TGCT remains unclear despite studies supporting a neoplastic mechanism rather than an inflammatory one [3]. Its clinical behaviour can range from indolent, localised forms to aggressive, recurrent lesions with destructive potential. TGCT is characterised by its aggressive clinical nature, resulting in a gradual deterioration of the affected joints [4].

The primary radiological observations, in cases of TGCT of the hip joint, involve the presence of multiple cyst-like or erosive areas in the upper part of the acetabulum, specifically in the non-weight-bearing regions of the acetabulum, the femoral neck, and the femoral head. The erosions and cyst-like lesions are extensive, affecting both the acetabulum and the femur. Due to the slow progression of TGCT, these lesions often exhibit thin sclerotic margins [5]. Currently, there are no approved medical drug treatments for TGCT. In most cases, the mainstay of management is surgical removal of the abnormal growth within or around the compromised joint [6,7]. A vast number of patients undergo arthroscopic and/or open surgical excision along with a synovectomy, but recurrence rates can be as high as 50% for those with diffuse or recurrent disease [8]. Joint arthroplasty is an effective treatment of choice for patients with hip joint TGCT. It has been proposed that opting for Total hip Arthroplasty (THA) results in reduced rates of disease recurrence and fewer revision procedures compared to synovectomy alone. This is primarily attributed to the predictable progression of joint deterioration in cases of diffuse TGCT [1,9].

## Case presentation

**Figure 1:**
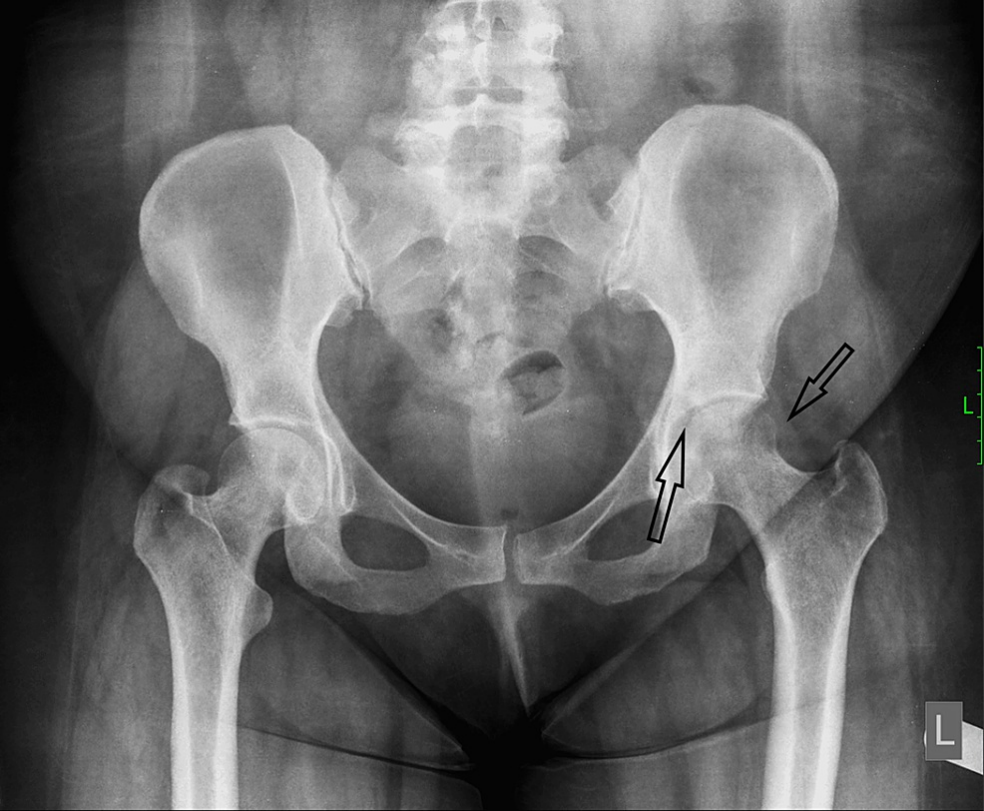
Preoperative anteroposterior radiographic view of the pelvis Rounded lucencies seen in the acetabulum and femoral head (both arrows)

A 39-year-old female patient presented to the orthopaedic department at Papageorgiou Hospital, Thessaloniki, with complaints of left hip pain and a three-month history of difficulty in walking. The patient had no history of weight loss or loss of appetite. She had no previous medical history and was under no medication. Upon examination, the patient had an antalgic gait. Her range of motion was slightly restricted due to pain. Νeurological evaluation was clear of any abnormal findings. X-rays showed osteolytic lesions both in the acetabulum and the femoral head (Figure 1).

**Figure 2:**
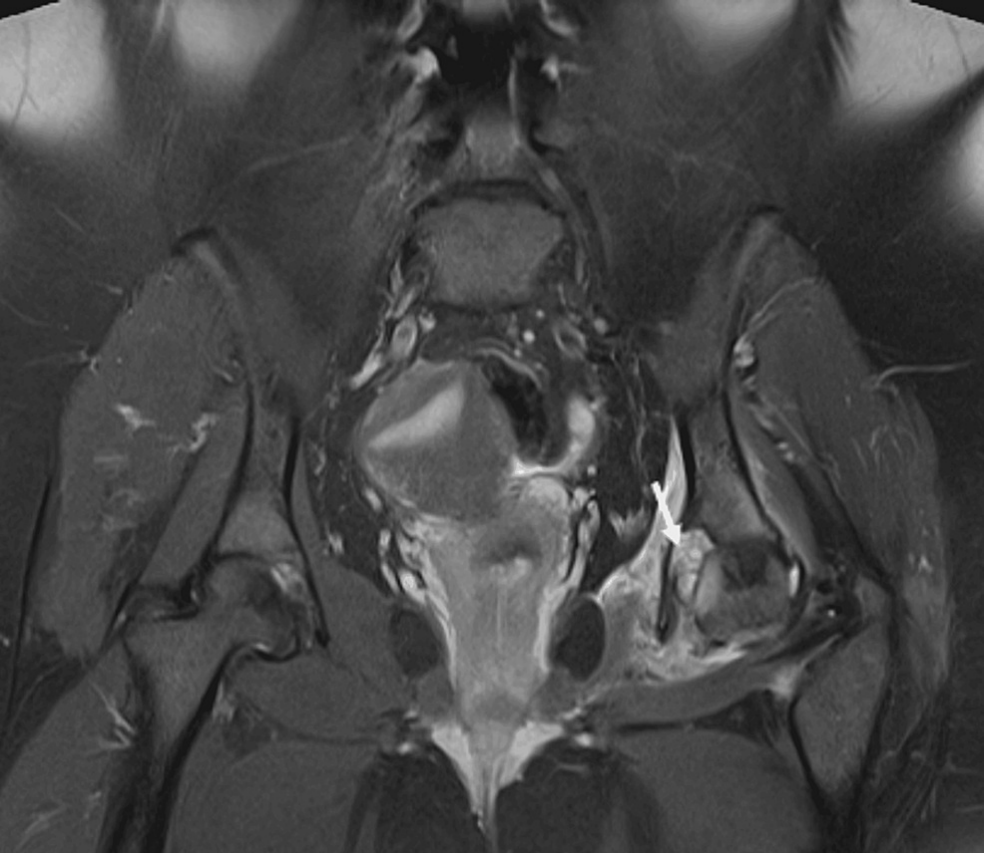
Coronal STIR sequence On the left fovea, there is a high-intensity, well-defined lesion (white arrow) STIR - Short tau inversion recovery

**Figure 3:**
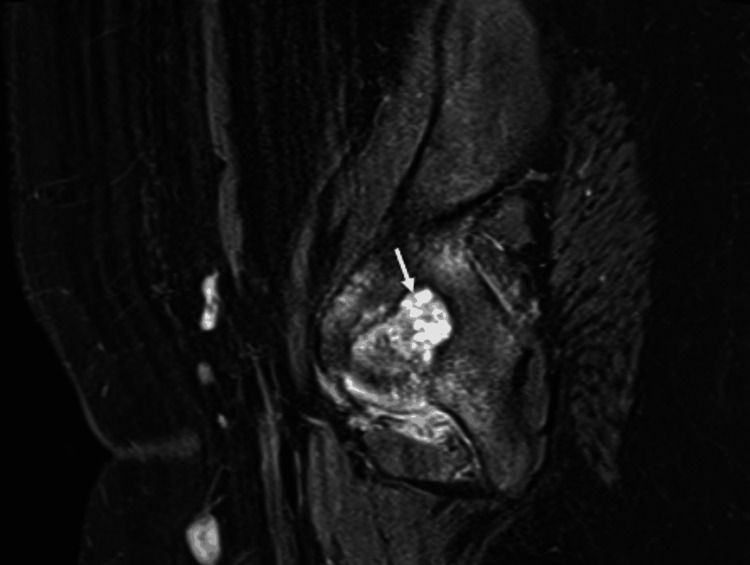
Sagittal PD with fat suppression sequence Extension of the lesion within the right fovea (white arrow)

**Figure 4:**
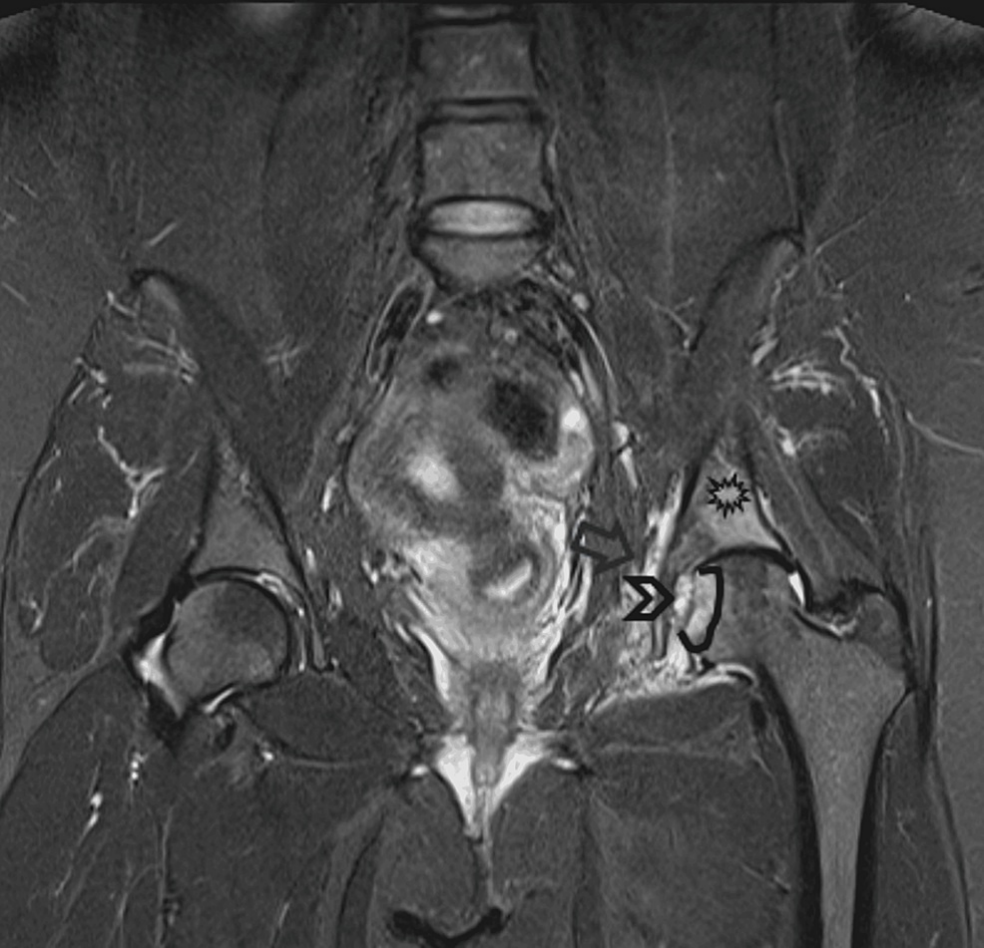
Coronal STIR sequence MRI Lesion (black open arrowhead) provokes intense bone oedema (asterisk), soft tissue oedema within the pelvis (black open arrow) as well as extension throughout the ligamentum teres towards the femoral head (black line) STIR - Short tau inversion recovery

**Figure 5:**
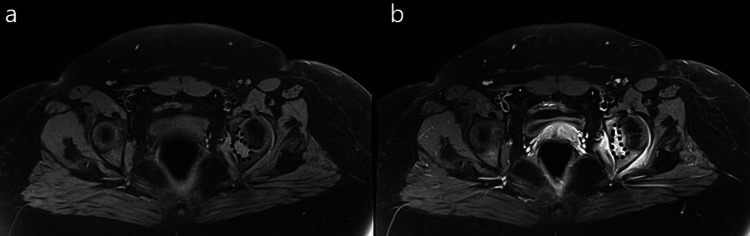
(a) Pre- and (b) post-contrast T1 with fat suppression images on axial plane Vivid enhancement of the lesion (black dot line) is depicted

MRI scan of the pelvis showed a well-defined lesion of high intensity on the left fovea in the coronal short tau inversion recovery (STIR) sequence (Figure 2). Sagittal proton density with fat suppression sequence displayed extension of the lesion within the right fovea (Figure 3). Another coronal STIR sequence revealed that the lesion provoked intense bone oedema, soft tissue oedema within the pelvis, and extension throughout the ligamentum teres in the femoral head (Figure 4). Pre- and post-contrast T1 with fat suppression images on the axial plane depicted vivid enhancement of the lesion (Figures 5a, 5b).

**Figure 6:**
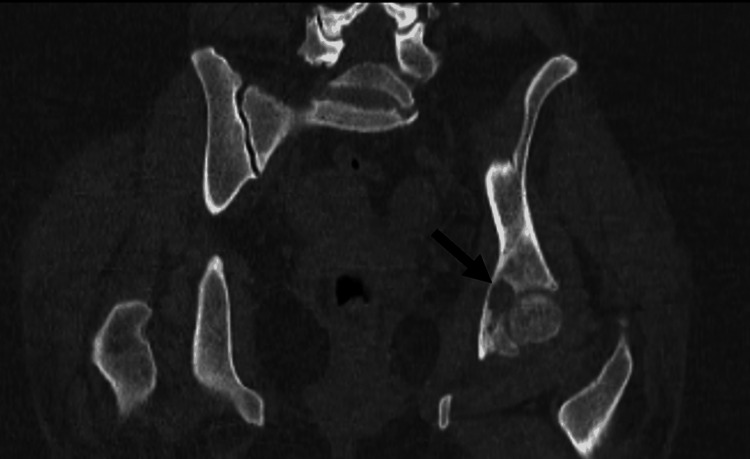
Coronal CT scan at the level of the lesion Lucent area within the left fovea (black arrow)

**Figure 7:**
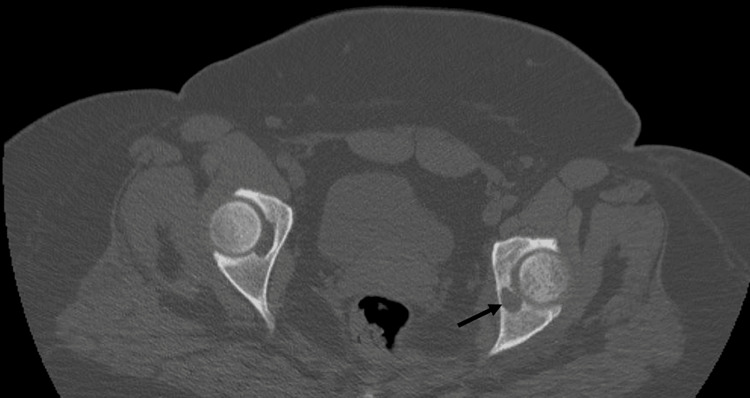
Axial CT scan at the level of the lesion Lucent area within the left fovea (black arrow)

A computed tomography (CT) scan revealed a thinning lesion in the middle of the acetabular joint, continuous with the fossa of the round ligament. More specifically, coronal (Figure 6) and axial (Figure 7) CT scans of the pelvis depicted a lucent area within the left fovea (black arrow). No nidus was identified within the lesion. A small amount of fluid was present in the left hip joint. There were also small and reactive inguinal lymph nodes.

**Figure 8:**
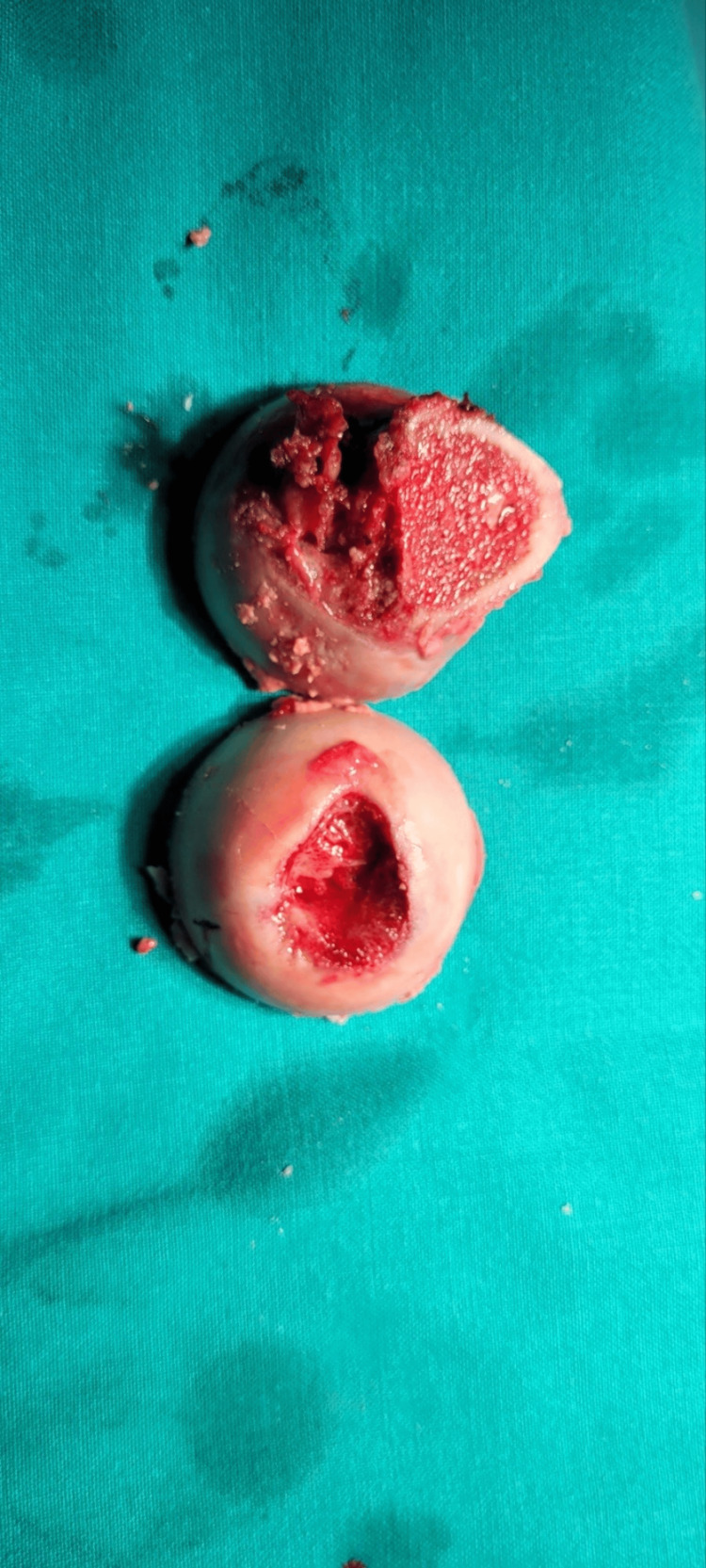
Resected femoral head Section of the femoral head demonstrating a deep central erosion (inferior hemisection).

**Figure 9:**
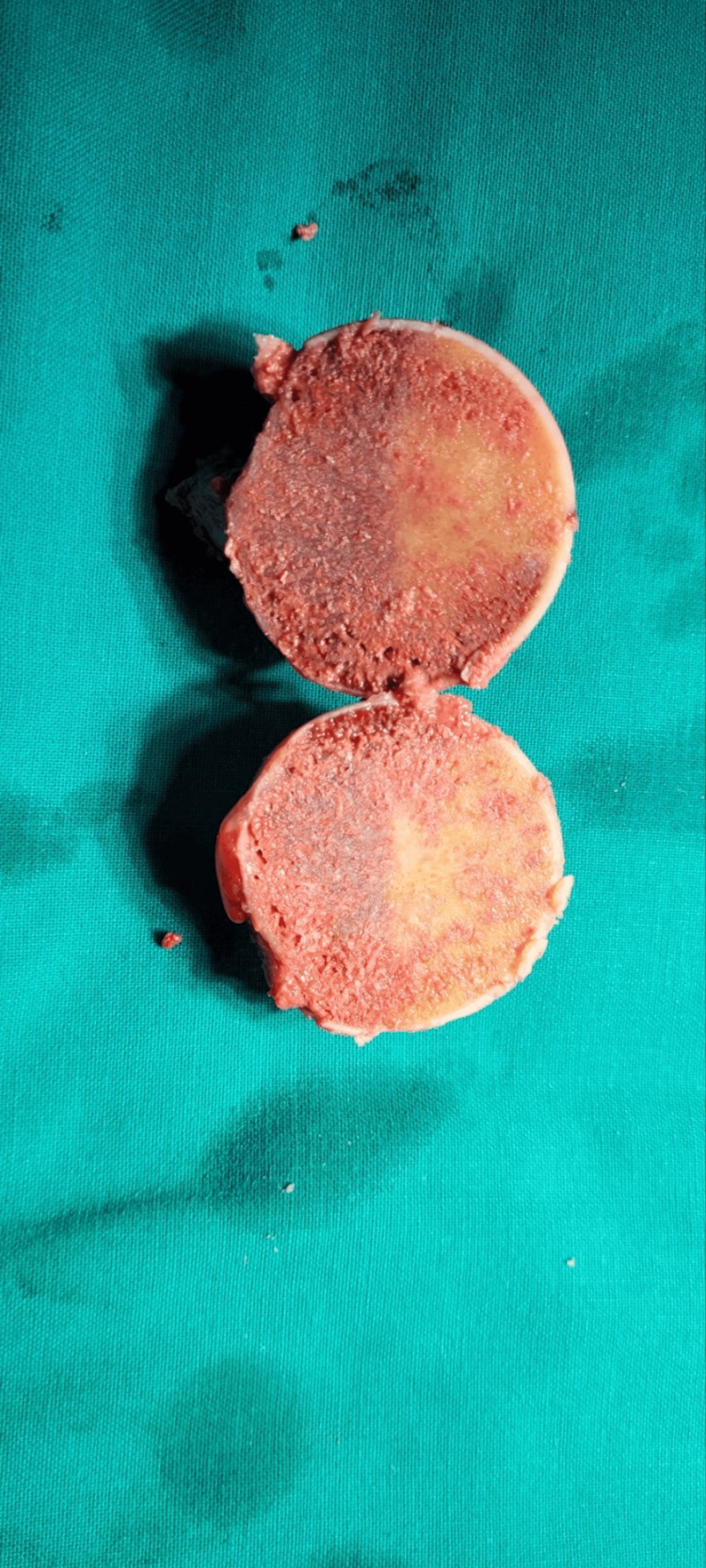
Hemisections of the femoral head Hemisections of the femoral head demonstrating pigmentation within the bone (superior hemisection)

**Figure 10:**
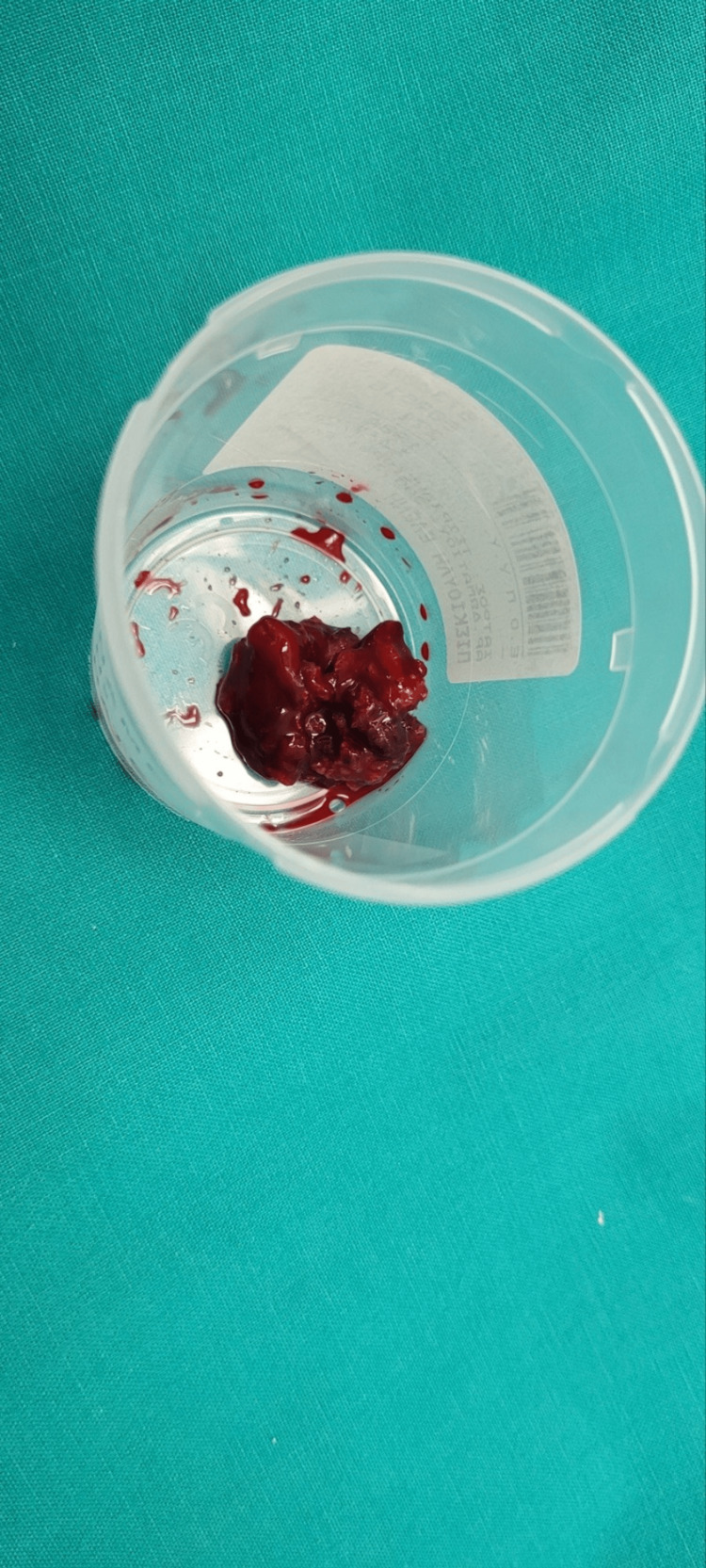
Firm and sponge-like lesion extracted from the acetabulum The removed lesion from the region of the acetabular fovea at the site of insertion of the ligamentum teres

**Figure 11:**
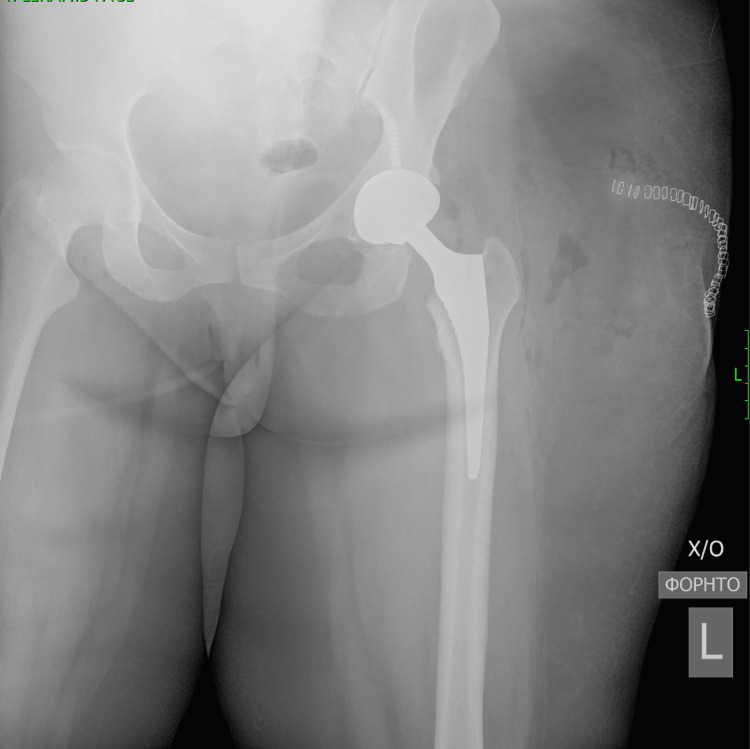
Post-operative anteroposterior radiographic view of the left hip

All tests were inconclusive. As there was no diagnosis, cementless THA was chosen as the most appropriate surgical option and was subsequently performed. The acetabular component used was U Motion II PS Cup Cluster Hole size 46 by United, the selected insert was U-Motion XPE Cup Liner (Highly Cross-linked Polyethylene) size 28 by United, the femoral stem used was Conformity Stem STD size 1 by United and Biolox Delta Ceramic 12/14 size 28 with +4 offset was the chosen femoral head. Intraoperatively, the lesion depicted in the scans was present from the acetabular fovea and migrating along the ligamentum teres, destroying the femoral head at the insertion of the ligamentum teres towards the middle of the femoral head (Figures 8-10). Following surgery, a biopsy identified the lesions as TGCT. Post-operative anteroposterior view of the left hip displayed excellent placement of both the femoral and acetabular components (Figure 11).

**Figure 12:**
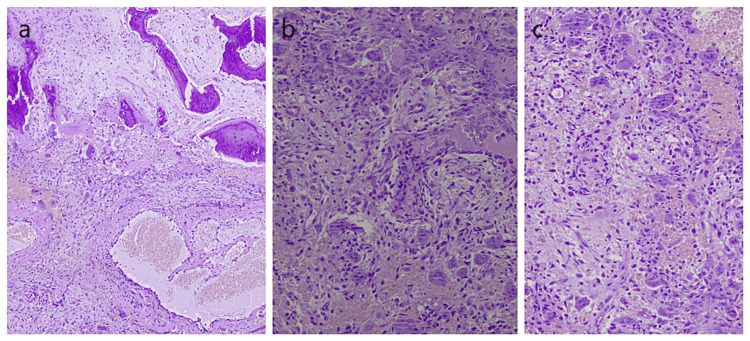
Histopathological examination (a-c) Multiple tissue sections derived from the histopathological examination demonstrated fragments of a mesenchymal-type process composed of mostly spindle-shaped cells growing within a fibrous or myxomatous substrate. Among them, numerous giant osteoclastic type cells, as well as areas of haemorrhagic necrosis with hemosiderin deposits were frequently identified. Additionally, osteoid deposits were observed.

Histological examination of the lesion revealed features consistent with TGCT (Figures 12a-12c).

The patient was scheduled for a follow-up appointment two months post-operatively. She was walking comfortably, with the help of a crutch, and she reported a significant decrease in pain. Hip joint range of motion had improved, with a flexion of 90°, an internal rotation of 10°, an external rotation of 15°, an abduction of 33° and an extension of 0°. Their hip disability and Osteoarthritis Outcome Score (HOOS) was 68.6 and her Harris Hip Score was 41.

## Discussion

Although uncommon, the growth of synovial tissue in patients with TGCT may result in painful erosion of cartilage and bone around the hip joint. The standard treatment approaches regarding erosive cartilage and bone in the area of the hip has commonly involved synovectomy, often paired with THA. Della et al. [9] documented seven cases of TGCTs. Among the four patients who underwent synovectomy along with THA, none of them experienced a recurrence of the disease 13-year post-op. Among the patients that underwent a synovectomy alone, one patient demonstrated no radiographic changes two years after the initial diagnosis, one exhibited severe bone loss after a 21-year radiographic follow-up, and one experienced a recurrence just nine months after the operation. Botez et al. [10] have provided insights into the recurrence rates of TGCT following synovectomy. The rates vary between 0% and 40% depending on the study. They also point out that the overall literature suggests a lower recurrence rate when THA is involved.

A recently published systematic review [11] examining a sample of 82 patient participants diagnosed with hip TGCT found a 17.8% recurrence rate when synovectomy alone was the preferred treatment option and a 3.8% recurrence rate when patients underwent synovectomy along with THA. However, it's worth noting that this difference was not statistically significant. Another study [12] involving 25 patients with histologically confirmed end-stage TGCT results showed that when THA is performed in the context of TGCT, patient functionality is enhanced while low local recurrence rates are maintained. Similarly, Yoo et al. [13] reported that out of eight patients, all had improved clinical results, and none had radiographic evidence of recurrent TGCT. Nevertheless, the incidence of complications and the need for revisions in both these studies was relatively high, possibly due to the youthful and active patient demographic, as well as the use of traditional polyethylene. The utilisation of modern bearing materials should reduce the risk of revision. Two critical factors influencing implant longevity are the following: method of implant fixation and type of bearing surface [14]. Ceramic on HXLPE bearing was chosen as there is strong evidence now that the wear rate is low for this type of articulation [12,15]. To date, there has not been any direct comparison of the different bearing surfaces in this patient group. Although there is limited data on TGCT of the hip, both cemented and cementless THA have been reported to yield favourable results.

## Conclusions

The case report emphasises the importance of considering TGCT as a diagnostic possibility when confronted with unexplained hip pain and inconclusive imaging findings while underscoring the effectiveness of cementless THA as a viable choice for TGCT patients. The procedure offers long-term pain relief and functional improvement. The variability in clinical presentation and diagnostic challenges should prompt clinicians to maintain a high index of suspicion and consider biopsy when radiological evidence is unclear.

In conclusion, this case adds to the limited body of literature on TGCT of the hip joint, highlighting the need for early recognition and personalised management approaches. Collaboration between orthopaedic surgeons, radiologists, and pathologists is essential to navigating the complexities regarding TGCT diagnosis and treatment.
